# Assessing the Effects of Non-steroidal Anti-inflammatory Drugs on Antihypertensive Drug Therapy Using Post-Marketing Surveillance Database

**DOI:** 10.2188/jea.JE2007413

**Published:** 2008-05-29

**Authors:** Chieko Ishiguro, Toshiharu Fujita, Takashi Omori, Yosuke Fujii, Takeshi Mayama, Tosiya Sato

**Affiliations:** 1 Office of Safety, Pharmaceutical and Medical Devices Agency; 2 Department of Data Science, The Institute of Statistical Mathematics; 3 Department of Biostatistics, Kyoto University School of Public Health; 4 RAD-AR (Risk/Benefit Assessment of Drugs-Analysis and Response) Council

**Keywords:** Drug Interactions, Antihypertensive Agents, NSAIDs, Database

## Abstract

**Background:**

Antihypertensive and non-steroidal anti-inflammatory drugs (NSAIDs) are used to treat many common diseases. However, it has been suspected that interactions between these drugs exist. Here, we assessed the interactions between non-selective NSAIDs and several classes of antihypertensive drugs.

**Methods:**

The study design was a cohort study using “The Antihypertensive Drug Database,” which is a collection of data accumulated from Drug Use Investigations. Subjects newly starting antihypertensive drug therapy were identified in the database. We compared the “User” group, who were co-administered NSAIDs, with the “Non-user” group, who were not. The outcome measure was the change in systolic blood pressure from the baseline after 2 months of treatment. We estimated the non-adjusted and adjusted differences in the change in systolic blood pressure between the “User” and “Non-user” groups.

**Results:**

Data were collected for a total of 1,204 subjects, of whom 364 were prescribed beta blockers, 60 were prescribed diuretics, 628 were prescribed angiotensin-converting enzyme inhibitors, and 152 were prescribed calcium channel blockers. The adjusted difference in the change in systolic blood pressure between the User (n = 301) and Non-user (n = 903) groups was 2.88 mmHg (95% confidence interval: 0.89, 4.87); thus, systolic blood pressure in the Non-User group decreased further from the baseline than that in the User group. In subjects administered beta blockers, diuretics, angiotensin-converting enzyme inhibitors, and calcium channel blockers, the corresponding differences were 0.37 mmHg (-3.24, 3.98), 6.11 mmHg (-3.16, 15.37), 3.85 mmHg (1.16, 6.66), and 3.50 mmHg (-2.03, 9.02).

**Conclusion:**

The effectiveness of antihypertensive drugs was attenuated by the co-administration of NSAIDs. The differences in the effects of NSAIDs varied with different classes of antihypertensive drugs.

## INTRODUCTION

Non-steroidal anti-inflammatory drugs (NSAIDs) are used all over the world for their analgesic, anti-inflammatory, and antipyretic effects. NSAIDs block the enzymatic activity of cyclooxygenase (COX) and lead to the inhibition of prostaglandin (PG) synthesis. Rofecoxib, released in 2000, was expected to improve gastrointestinal side effects by selectively blocking COX2, one of the COX isoenzymes, but was withdrawn in 2004 because of its cardiovascular risks.^1^ Regulatory agencies in the European Union and the United States now consider this problem to be specific not only to selective COX2 inhibitors, but also to non-selective NSAIDs.^2^^,^^3^

While non-selective NSAIDs have been reported to increase blood pressure in individuals with hypertension, particularly among users of beta blockers (BBs),^4^ it has been suggested that physicians do not recognize this effect of NSAIDs.^5^ Almost all major classes of antihypertensive drugs, with the possible exception of calcium channel blockers (CCBs), exert all or part of their therapeutic actions through PG-mediated mechanisms.^6^ NSAIDs, by interfering with PG synthesis, may thus limit the ability of these drugs to control blood pressure.^6^ Pharmacologically, it is thought that NSAIDs interact differently with antihypertensive drugs. However, the effects of NSAIDs on newly initiated antihypertensive drug therapy remain unclear because few studies have included patients who were initially administered NSAIDs and then antihypertensives.

In Japan, non-selective NSAIDs are used widely because no specific COX2 inhibitors were approved until January 2007. In the elderly population (individuals aged 65 years and older), hypertension is the most common disease, while arthritis is ranked fourth, according to the Patient Survey by the Japanese Ministry of Health, Labour and Welfare.^7^ Many people are thought to have simultaneously consumed antihypertensive drugs and NSAIDs. Small increases in systolic blood pressure (SBP) over time are linked to meaningful increases in coronary heart disease, stroke, and death in older populations.^5^ Therefore, appropriate antihypertensive treatments need to be identified for these NSAID users.

We conducted a cohort study using a database to estimate the effects of NSAIDs on antihypertensive drug therapy as well as potential differences between the different classes of antihypertensive drugs.

## METHODS

### Database

The Anti-Hypertensive Drugs Database from Post-Marketing Surveillance was developed by the Risk/Benefit Assessment of Drugs-Analysis and Response (RAD-AR) Council of Japan.^8^^,^^9^ It combines information on subjects participating in the Drug Use Investigation (“*shiyo seiseki chosa*”) conducted for Japanese Drug Reexamination Applications (“*sai shinsa*”) by every pharmaceutical manufacturer, in conformity with the Japanese Pharmaceutical Affairs Law (“*yakuji ho*”) and related regulations.^10^ This anonymous database contains data on 125,657 subjects taking antihypertensive drugs from 19 Drug Use Investigations (6 angiotensin-converting enzyme inhibitors (ACEIs), 4 CCBs, 6 BBs, 2 alpha blockers, and 1 diuretic) between 1981 and 1999. The present study protocol was approved by the boards of the RAD-AR Council.

### Subjects

Individuals with essential hypertension who did not have a history of exposure to antihypertensive drugs before the onset of antihypertensive therapy were identified from the database. Of the 34,006 subjects who met this criterion, 11,699 were excluded because of incomplete information regarding concomitant drugs and blood pressure. The study cohort eligible for analysis thus consisted of 22,307 subjects. The “User” group was defined as 301 subjects who concomitantly used NSAIDs other than low-dose aspirin and acetaminophen. Considering the operability of matching and statistical precision, we randomly selected 3 age-, sex-, and Drug Use Investigation-matched subjects for each “User” from those who were not exposed to NSAIDs in the eligible population and defined 903 subjects as “Non-users.”

### Effects of Antihypertensive Drug Therapy

In elderly people, as SBP increases steadily with age, while diastolic blood pressure (DBP) declines, the prevalence of systolic hypertension increases with age. Furthermore, SBP is more potent than DBP as a cardiovascular disease risk factor.^12^^,^^13^ It is known that changes in treatment regimen are indicated if patients do not achieve their treatment goals within 2 or 3 months after initiating a new therapy.^13^ In this study, therefore, the outcome measure of the effects of antihypertensive therapy was the change in SBP from the baseline after 2 months (±2 weeks).

### Analysis

Baseline characteristics were tabulated for overall comparison between the User and Non-user groups, and for each individual class of antihypertensive drug. The crude differences between the User and Non-user groups were calculated by subtracting the mean change in the SBP at 2 months post-treatment from the baseline SBP in the User group from this value in the Non-user group.

In primary analysis, multiple regression analysis was performed to adjust for covariates such as sex, age, classification of hypertension by extent of organ damage,^14^^,^^15^ baseline SBP, Drug Use Investigation, use of medications that influence hypertension (i.e., estrogens, corticosteroids, sympathomimetics, antihypertensive drugs, antidepressants, anticoagulants and coronary vasodilators), and complications (i.e., diabetes, hyperlipidemia, cerebrovascular disease, renal disease, arrhythmia, ischemic heart disease, heart failure and other forms of heart disease). The medication and complication covariates were dichotomous variables (0 or 1).

This standard regression model assumed a linear model to adjust for the confounding of multiple continuous and discrete covariates. In order to validate the linear model, we reanalyzed the effects of NSAIDs on antihypertensive therapy by using a semiparametric regression model^16^ (a type of propensity score analysis) to adjust for confounding. This was carried out by modeling the conditional expectations of NSAID exposure given the confounding variables, which is known as the propensity score

In order to investigate the differences in the effects of NSAIDs on antihypertensive drug therapy among different classes of medication, we also used a model with interaction terms between NSAIDs and the class of antihypertensive drug in the multiple regression analysis. We also preformed multiple regression analyses within subgroups defined by the class of antihypertensive drug for sensitivity analysis. In addition, we performed another multiple regression analysis within the subgroups defined in the Drug Use Investigations, in order to identify additional relationships.

In the analyses described above, in some subjects, information regarding the duration of concomitant drug administration did not exist. As sensitivity analysis to estimate the effect of the missing information, we performed multiple regression analysis within subgroups defined by the existence or nonexistence of information on dosing periods of NSAIDs, which was no later than the date of outcome measurement. We also carried out a similar analysis in the subgroup of patients receiving only 1 type of antihypertensive drug by excluding those co-prescribed several types of antihypertensive drugs.

Statistical analyses were conducted using JMP^®^4.0J and SAS^®^ Ver. 8.02.

## RESULTS

A total of 1,204 subjects in 11 Drug Use Investigations were selected: 324 of these received BBs, 60 received diuretics, 628 received ACEIs, and 152 received CCBs. Baseline information for the User and Non-user groups are summarized in Table 1. Subjects were similar across all groups with respect to these characteristics. A total of 64.1% of the subjects were female, and 50% were aged 65 years or older.

**Table 1.** Baseline characteristics of all subjects.

	Total (%)		Beta blockers (%)		Diuretics (%)		ACE inhibitors (%)		Calcium channel blockers (%)	

	User^*^	Non-user^†^	User	Non-user	User	Non-user	User	Non-user	User	Non-user
n	301	903	91	273	15	45	157	471	38	114
Female	193 (64)	579 (64)	61 (67)	183 (67)	9 (60)	27 (60)	101 (64)	303 (64)	22 (57)	66 (58)
Age^‡^	63.3 ± 12.5	63.3 ± 12.5	60.1 ± 13.4	60.6 ± 13.5	59.3 ± 11.4	58.2 ± 11.6	65.5 ± 11.8	65.5 ± 11.9	63.6 ± 12.1	64.2 ± 12.0
Systolic blood pressure^‡^	172.2 ± 15.5	171.7 ± 17.0	172.0 ± 15.9	172.3 ± 17.2	169.2 ± 15.1	171.7 ± 16.8	172.7 ± 15.7	171.1 ± 17.0	171.9 ± 10.9	172.7 ± 16.6
Classification of hypertension^§^										
1	207 (69)	596 (67)	60 (66)	173 (65)	7(47)	21 (51)	112 (72)	326 (71)	28 (76)	76 (67)
2	70 (23)	225 (25)	24 (26)	73 (27)	5 (33)	18 (44)	34 (22)	101 (22)	7 (19)	33 (29)
3	22 (7)	63 (7)	7 (8)	21 (8)	3 (20)	2 (5)	10 (6)	35 (8)	2 (5)	5 (4)
missing	2 (1)	19 (2)	0 (0)	6 (2)	0 (0)	4 (9)	1 (1)	9 (2)	1 (3)	0 (0)

ACE inhibitors: angiotensin-converting enzyme inhibitors

* : Those co-administered antihypertensive and nonsteroidal anti-inflammatory drugs

† : Those not co-administered antihypertensive and nonsteroidal anti-inflammatory drugs

‡ : Mean ± standard deviation

§ : Classification of hypertension by extent of organ damage

Table 2 shows the concomitant drugs used that caused refractory hypertension^17^ and complications. Antidepressants were used most commonly. Sympathomimetics included only bronchodilators (not appetite suppressants, topical vasoconstrictors, or isotropic agents). The prevalence of corticosteroid administration was higher in the User group (5%) than in the Non-user group (0%). Co-prescriptions for antihypertensive drugs accounted for 23% of the total. As expected, arthritis, including osteoarthritis and rheumatoid arthritis, was considerably more common in the User group (16%) than in the Non-user group (0.1%). Complications and the medications used were similar across all classes of antihypertensive drugs.

**Table 2.** Concomitant drugs and complications.

	Total (%)		Beta blockers (%)		Diuretics (%)		ACE inhibitors (%)		Calcium channel blockers (%)	

	User	Non-user	User	Non-user	User	Non-user	User	Non-user	User	Non-user
Concomitant drugs										
Antidepressants	23 (8)	16 (2)	11 (12)	5 (2)	0 (0)	0 (0)	10 (6)	8 (2)	2 (5)	3 (3)
Corticosteroids	14 (5)	4 (0.4)	5 (5)	1 (0)	1 (7)	0 (0)	5 (3)	1 (0.2)	3 (8)	2 (2)
Sympathomietics	3 (1)	6 (1)	2 (2)	0 (0)	0 (0)	0 (0)	0 (0)	3 (1)	1 (3)	3 (3)
Co-prescriptions with antihypertensive drugs										
Total	54 (18)	221 (24)	24 (26)	78 (29)	1 (7)	12 (27)	26 (17)	116 (25)	3 (8)	15 (13)
Beta blockers	5 (2)	32 (4)	1 (1)	4 (1)	1 (7)	4 (9)	3 (2)	19 (4)	0 (0)	5 (4)
alpha blockers	6 (2)	11 (1)	1 (1)	4 (1)	1 (7)	4 (9)	3 (2)	19 (4)	0 (0)	5 (4)
Diuretics	5 (2)	26 (3)	1 (1)	5 (2)	0 (0)	1 (2)	4 (3)	18 (4)	0 (0)	2 (2)
ACE inhibitors	12 (4)	31 (3)	10 (11)	20 (7)	0 (0)	2 (4)	0 (0)	2 (0.5)	2 (5)	7 (6)
Calcium channel blockers	29 (10)	142 (16)	12 (13)	54 (20)	1 (7)	4 (9)	15 (10)	83 (18)	1 (3)	1 (1)
Others	3 (1)	12 (1)	0 (0)	5 (2)	1 (7)	2 (4)	2 (1)	5 (1)	0 (0)	0 (0)
Anticoagulants	12 (4)	34 (4)	3 (3)	9 (3)	0 (0)	0 (0)	8 (5)	16 (3)	1 (3)	9 (8)
Coronary vasodilators	21 (7)	42 (5)	4 (4)	15 (5)	1 (7)	2 (4)	8 (5)	18 (4)	8 (21)	7 (6)

Complications										
Ischemic heart disease	14 (5)	48 (5)	5 (5)	20 (7)	0 (0)	0 (0)	7 (4)	24 (5)	2 (5)	4 (4)
Diabetes	30 (10)	87 (10)	7 (8)	25 (9)	3 (20)	1 (2)	16 (10)	46 (10)	4 (11)	15 (13)
Hyperlipidemia	27 (9)	114 (13)	7 (8)	41 (15)	0 (0)	0 (0)	18 (11)	61 (13)	2 (5)	12 (11)
Celebrovascular disease	24 (8)	75 (8)	3 (3)	17 (6)	4 (27)	2 (4)	16 (10)	45 (10)	1 (3)	11 (10)
Other forms of heart disease	13 (4)	44 (5)	4 (4)	11 (4)	2 (13)	5 (11)	7 (4)	27 (6)	0 (0)	1 (1)
Arrhythmia	8 (3)	23 (3)	1 (1)	9 (3)	1 (7)	0 (0)	6 (4)	9 (2)	0 (0)	5 (4)
Heart failure	1 (0.1)	7 (1)	0 (0)	0 (0)	0 (0)	0 (0)	1 (1)	7 (1)	0 (0)	0 (0)
Renal disease	1 (0.1)	6 (1)	1 (1)	2 (1)	0 (0)	1 (2)	0 (0)	1 (0.2)	0 (0)	2 (2)
Arthritis	49 (16)	1 (0)	11 (12)	0 (0)	0 (0)	1 (0)	28 (18)	0 (0)	10 (26)	0 (0)

ACE inhibitors: angiotensin-converting enzyme inhibitors

Table 3 shows the mean change in SBP from the baseline to the 2-month time point and the crude and adjusted differences in the mean change in SBP between the User and Non-user groups. For all the subjects, the crude and adjusted differences were 1.96 mmHg (95% confidence interval [CI]: -0.53, 4.48) and 2.88 mmHg (95% CI: 0.89, 4.87) respectively, confirming that SBP decreased further from the baseline in the Non-user group than in the User group. The results obtained using the semiparametric model showed a 2.87 mmHg difference in the User group, which was virtually the same result as that obtained with the standard linear model.

**Table 3.** Differences in the effect of antihypertertensive drug therapy on systolic blood pressure in Users^*^ and Non-users.^†^

	Total	Beta blockers	Diuretics	ACE inhibitors	Calcium channel blockers
Change in systolic blood pressure^‡^ (mmHg)					
User^*§^	-24.2 ± 18.9	-27.3 ± 20.9	-18.8 ± 17.9	-22.4 ± 17.4	-26.3 ± 19.3
Non-user^†§^	-26.2 ± 19.3	-27.8 ± 19.6	-24.4 ± 16.6	-24.6 ± 19.5	-29.6 ± 18.1

Difference in the change in systolic blood pressure (mmHg)					
crude	1.98	0.45	5.62	2.19	3.30
(95% CI)	(-0.53, 4.48)	(-4.30, 5.20)	(-4.47, 15.71)	(1.24, 5.63)	(-3.52, 10.12)
adjusted^||^	2.88	0.37	6.11	3.85	3.50
(95% CI)	(0.89, 4.87)	(-3.24, 3.98)	(-3.16, 15.37)	(1.16, 6.66)	(-2.03, 9.02)

ACE: inhibitors: angiotensin-converting enzyme inhibitors

CI: confidence interval

* : Those co-administered antihypertensive and nonsteroidal anti-inflammatory drugs

† : Those not co-administered antihypertensive and nonsteroidal anti-inflammatory drugs

‡ : Change in the baseline systolic blood pressure after 2 months

§ : Mean ± standard deviation

|| : Adjusted for sex, age, classification of hypertension by extent of organ damage baseline systolic blood pressure, Drug Use Investigation, the use of medications that influence hypertension, and complications

For each class of antihypertensive drug, the adjusted differences were obtained using interaction terms in the multiple regression analysis. In the group taking BBs, no differences were noted (0.37 mmHg, 95% CI: -3.24, 3.98). The group treated with diuretics showed the largest difference at 6.11 mmHg (95% CI: -3.16, 15.37). In the group taking ACEIs, the difference was 3.85 mmHg (95% CI: 1.16, 6.55), and the difference in the CCB group was 3.50 mmHg (95% CI: -2.03, 9.02). The other results from multiple regression analyses within subgroups defined by the class of antihypertensive drug were similar to the results obtained using interaction terms.

We also performed multiple regression analysis within the subgroups defined in the Drug Use Investigations. BBs were investigated in 4 different Drug Use Investigations, and the differences were -0.42, -0.64, 3.93, and 5.21 mmHg. ACEIs were studied in 5 Drug Use Investigations, and the differences were 0.37, 4.88, 3.47, 1.58, and 6.49 mmHg. Diuretics and CCBs were not separately studied in Drug Use Investigations because there was only one Drug Use Investigation for each.

Another multiple regression analysis was performed to assess the effect of missing information regarding the duration of concomitant drug administration. The results revealed that the adjusted difference between the User and Non-user groups in the subgroup that had information was 3.95 mmHg (95% CI: 1.36, 6.54). The adjusted difference in the group receiving only 1 type of antihypertensive medication was 2.23 mmHg (95% CI: 0.10, 4.36).

## DISCUSSION

We observed in the primary analysis that SBP decreased further from the baseline in the Non-user group than in the User group and the difference between the Non-user and User groups was 2.88 mmHg. This is very important because it has been reported that a decrease of 2.2 mmHg in SBP lowers mortality due to coronary heart disease by 4%.^18^ In past studies, NSAIDs have been reported to increase blood pressure in subjects treated first with antihypertensive agents.^4^ Our results support this and suggest that the effects of antihypertensive drugs are also attenuated in subjects initially or simultaneously treated with NSAIDs.

With regard to individual classes of antihypertensive drugs, our results showed that diuretics, ACEIs, and CCBs are affected by NSAIDs, whereas BBs are not. Physiologically, the effects of renal PGs on salt and water transport in the kidney are complementary to the actions of diuretics. Therefore, it is likely that the blocking of PG synthesis by NSAIDs attenuates the effect of diuretics.^6^ ACEIs produce vasodilatation and lower blood pressure by inhibiting ACE, which promotes the formation of angiotensin-2 and aldosterone. Bradykinin is an autacoid that produces vasodilatation and further reduces blood pressure. Blocking ACE decreases the inhibition of bradykinin-induced vasodilatation. However, the vasodilatory properties of bradykinin that contribute to the antihypertensive properties of ACE inhibition appear to be mediated through local release of PGs and are therefore susceptible to interference by NSAIDs.^19^ The results of this study are thus consistent with pharmacological expectations concerning the action of diuretics and ACEIs.

On the other hand, CCBs do not depend on vascular PG production as a part of their mechanism of action.^6^ Clinical studies have reported that CCBs are less affected by NSAIDs than ACEIs.^20^^,^^21^ In our study, however, the results for ACEIs and CCBs were nearly identical. The confidence intervals are quite wide because sample sizes in the diuretic and CCB groups were much smaller than those in the other groups.

Although there may be residual confounding, the effects of these different drug classes remained unclear in this study. Non-selective NSAIDs have been reported to increase blood pressure in individuals with hypertension, particularly among those using BBs.^4^ Blood pressure reduction by BBs is partially attributable to the inhibition of rennin secretion, but other mechanisms, many of which have not yet been clarified in detail, also play a role.^19^ It is possible that NSAIDs block the antihypertensive action of BBs since propranolol reportedly stimulates PG synthesis in patients with essential hypertension, and BBs are reportedly inhibited by NSAIDs.^22^ In our study, however, BBs were largely unaffected by NSAIDs.

The results of multiple regression analysis within the subgroups defined in the Drug Use Investigations revealed that in subjects who used BBs, qualitative interactions between NSAIDs and Drug Use Investigations were present. The discrepancy concerning BBs in this study may not be due to the mechanism of action but rather due to the differences in the methods of investigation or the products themselves since each Drug Use Investigation was conducted separately by each pharmaceutical manufacturer for each product. On the other hand, in subjects using ACEIs, we found quantitative interactions and that the effects of ACEIs were consistently attenuated by NSAIDs.

This study has other limitations, most notably that several variables of interest were not available in the study database, including body weight and height, tobacco use, the method of blood pressure measurement, and laboratory results other than SBP. Furthermore, imperfect information regarding the duration of concomitant drug administration was a major limitation of this study. In the primary analysis in this study, we selected subjects regardless whether information on the dosing periods of NSAIDs was present or absent because information on concomitant drugs was based on data gathered upon enrolment in the Drug Use Investigations. However, for 44% of the User group, the database contained only the drug codes of concomitantly administered NSAIDs and lacked information concerning the period of their use. To estimate the effect of this missing information, we performed subgroup analysis as a sensitivity analysis. The adjusted difference between the User and Non-user subgroups with the information was 3.95 mmHg, indicating the attenuation of antihypertensive effect by combined medication with NSAIDs. The adjusted difference in the primary analysis, 2.88 mmHg might be underestimated because of the selection regardless of the missing information.

As prescribing multiple antihypertensive drugs is common during the course of therapy, it is important to assess the effects of NSAIDs on treatment with a single or with multiple antihypertensive drugs. Because of imperfect information regarding the period of administration of concomitantly used antihypertensive drugs other than the first antihypertensive drug given, we analyzed the mono-medication group. The adjusted difference in the mono-medication group was 2.23 mmHg, which is similar to the result of the primary analysis. Although imperfect information on the dosing period of concomitant drugs remains a limitation of this study, the results from these sensitivity analyses suggest the attenuation of antihypertensive effect by combined medication with NSAIDs. Another consideration is how different classes of NSAIDs affect antihypertensive drug therapy. Although such an investigation was undertaken with imperfect information on the dose and duration and the various NSAIDs, the distribution of NSAID classes used by subjects was similar among the 4 User groups.

In conclusion, the effectiveness of antihypertensive drugs was attenuated by co-administration of NSAIDs. However, the differences in the effects of NSAIDs on different classes of antihypertensive drugs were not clarified because in the BB group, the effects of NSAIDs varied among 4 Drug Use Investigations and in the diuretic and CCB groups, the sample sizes were small. Further investigations are required to evaluate the effects of co-prescribing antihypertensive drugs and NSAIDs.
